# Notch-Jagged1 signaling and response to bevacizumab therapy in advanced colorectal cancer: A glance to radiomics or back to physiopathology?

**DOI:** 10.3389/fonc.2023.1132564

**Published:** 2023-02-28

**Authors:** Francesca Negri, Lorena Bottarelli, Giuseppe Pedrazzi, Michele Maddalo, Ludovica Leo, Gianluca Milanese, Roberto Sala, Michele Lecchini, Nicoletta Campanini, Cecilia Bozzetti, Andrea Zavani, Gianluca Di Rienzo, Cinzia Azzoni, Enrico Maria Silini, Nicola Sverzellati, Federica Gaiani, Gian Luigi de’ Angelis, Letizia Gnetti

**Affiliations:** ^1^ Gastroenterology and Endoscopy Unit, University Hospital of Parma, Parma, Italy; ^2^ Pathology Unit, Department of Medicine and Surgery, University of Parma, Parma, Italy; ^3^ Department of Medicine and Surgery, University of Parma, Parma, Italy; ^4^ Medical Physics Department, University Hospital of Parma, Parma, Italy; ^5^ Radiology, Department of Medicine and Surgery, University of Parma, Parma, Italy; ^6^ Oncology Unit, University Hospital of Parma, Parma, Italy; ^7^ Pathology Unit, University Hospital of Parma, Parma, Italy

**Keywords:** Notch signaling pathway, bevacizumab, colorectal cancer, Jagged-1, therapy resistance

## Abstract

**Introduction:**

The Notch intracellular domain (NICD) and its ligands Jagged-1(Jag1), Delta-like ligand (DLL-3) and DLL4 play an important role in neoangiogenesis. Previous studies suggest a correlation between the tissue levels of NICD and response to therapy with bevacizumab in colorectal cancer (CRC). Another marker that may predict outcome in CRC is radiomics of liver metastases. The aim of this study was to investigate the expression of NICD and its ligands and the role of radiomics in the selection of treatment-naive metastatic CRC patients receiving bevacizumab.

**Methods:**

Immunohistochemistry (IHC) for NICD, Jag1 and E-cadherin was performed on the tissue microarrays (TMAs) of 111 patients with metastatic CRC treated with bevacizumab and chemotherapy. Both the intensity and the percentage of stained cells were evaluated. The absolute number of CD4+ and CD8+ lymphocytes was counted in three different high-power fields and the mean values obtained were used to determine the CD4/CD8 ratio. The positivity of tumor cells to DLL3 and DLL4 was studied. The microvascular density (MVD) was assessed in fifteen cases by counting the microvessels at 20x magnification and expressed as MVD score. Abdominal CT scans were retrieved and imported into a dedicated workstation for radiomic analysis. Manually drawn regions of interest (ROI) allowed the extraction of radiomic features (RFs) from the tumor.

**Results:**

A positive association was found between NICD and Jag1 expression (p < 0.001). Median PFS was significantly shorter in patients whose tumors expressed high NICD and Jag1 (6.43 months vs 11.53 months for negative cases; p = 0.001). Those with an MVD score ≥5 (CD31-high, NICD/Jag1 positive) experienced significantly poorer survival. The radiomic model developed to predict short and long-term survival and PFS yielded a ROC-AUC of 0.709; when integrated with clinical and histopathological data, the integrated model improved the predictive score (ROC-AUC of 0.823).

**Discussion:**

These results show that high NICD and Jag1 expression are associated with progressive disease and early disease progression to anti VEGF-based therapy; the preliminary radiomic analyses show that the integration of quantitative information with clinical and histological data display the highest performance in predicting the outcome of CRC patients.

## Introduction

Notch signaling is an evolutionary conserved pathway that plays a critical role in regulating cell-fate differentiation during embryonic development (1, 2). This pathway also affects angiogenesis (3), is aberrantly activated in several cancers and influences malignant proliferation and progression (4). The activation of the Notch pathway arises when specific ligands, such as Jagged-1 (Jag1) or Delta-like ligand (DLL)-3 or DLL4, bind to the Notch transmembrane receptor (1). Jag1 or DLL ligand binding to Notch receptor leads to the separation of the Notch extracellular domain by proteases of the ADAM family. Subsequently, the Notch intracellular domain (NICD) is released by a gamma-secretase processing and transits to the nucleus where it regulates downstream gene expression (1).

Notch signaling triggered *via* Jag1 and DLLs plays a double role (5, 6): it inhibits DLLs (5) while it activates Jag1 (6). Previous studies have revealed that Notch signaling can be triggered by soluble forms of DLLs and Jag1 (7–9), which have different consequences on tumor progression: while soluble DLLs hinder tumor growth (10), soluble Jag1 greatly exacerbates the malignant development of cancer. Jag1 plays a key role in promoting epithelial to mesenchymal transition (EMT) as well as fostering cancer stem cell (CSC) phenotypes (8). Our previous data suggested an association between high tissue levels of NICD and poorer response to anti-vascular endothelial growth factor (VEGF) bevacizumab as first-line therapy in metastatic colorectal cancer (CRC) patients, but not to chemotherapy alone (11). No association was found between NICD and DLL4 expression within the same tumor (11). Jag1 might reduce Notch signaling, thereby enhancing responses to VEGF; such tumors could therefore be more susceptible to VEGF inhibition or different anti-angiogenic treatments.

The role of imaging in CRC staging has been recently expanded by the implementation of non-invasive biomarkers extrapolated from medical images (12). Radiomics of liver metastases in patients with CRC showed to predict outcome in patients treated with FOLFIRI and bevacizumab (13). Recent attention has been given to a multiomics strategy for comprehensive genotype–phenotype characterization of several oncological diseases (14, 15). Proteomics analysis can uncover new therapeutic choices, thus reducing the emergence of drug resistance and potentially improving patient outcomes (16). However, research mostly focused on radiomics alone, without attempting to integrate the radiomic signature with reliable clinical predictors and molecular data (KRAS mutation status or microsatellite instability) (17). Therefore, predictive models in CRC patients might be further improved by multidisciplinary approaches encompassing quantitative metrics derived from diagnostic studies, which have been more widely used for other cancer types, instead.

These data prompted us to investigate the expression of NICD, Jag1, DLL3 and DLL4 and a series of markers potentially involved in angiogenesis and immune response to bevacizumab therapy.

We also tested whether radiomics could select treatment-naive metastatic CRC patients responding to bevacizumab, beyond clinical and NICD/Jag1/DLL expression parameters.

## Materials and methods

We characterized a series of tumors by using immunohistochemistry (IHC) in tissue microarrays (TMAs) from 111 pre-treatment surgical specimens from patients with metastatic CRC treated with anti VEGF-therapy bevacizumab in combination with chemotherapy between 2008 and 2017 at the University Hospital of Parma (Parma, Italy). Cases were selected based on the availability of retrospective archival-FFPE (formalin-fixed, paraffin-embedded) tissue specimens. The study protocol was approved by the local Ethics Committee (AVEN: Comitato Etico dell’Area Vasta Emilia Nord). The procedures used in this study adhere to the tenets of the Declaration of Helsinki. Response to bevacizumab was assessed by using time point RECIST version 1.1 (i.e. best response at time point).

NICD staining and other parameters were collected; patients’ demographics, primary tumor characteristics and therapy details are listed in **Table 1**.

**Table 1:** Patients’ characteristics and tissue microarray expression data.

Characteristics	N = 111 (%)
Age (years)	66
Range	32-84
Sex	
Male	60 (54)
Female	51 (46)
CEA	
<30	57 (51)
>30	41(37)
Unknown	13 (12)
Primary tumor side	
Right side	47 (43)
Left side	63 (57)
Unknown	1
Number of metastatic sites	
1	51 (46)
≥2	60 (54)
Subsequent chemotherapy	
Yes	87 (79)
Received aflibercept	9/87 (10)
KRAS	
Mutant	61 (68)
Wild type	29 (32)
Unknown	21
NICD	
High	42 (38)
Low	69 (62)
Jag1	
Positive	68 (62)
Negative	42 (38)
DLL4	
Positive	90 (86)
Negative	14 (14)
Not evaluable	7
DLL3	
Positive	79 (81)
Negative	18 (19)
Not evaluable	14
CD4/CD8	
2/1-3/1 1/1	84 (92) 6 (7)
1/2	1 (1)
Not evaluable	20
CD3	
Positive	94 (100)
Negative	0
Not evaluable	17
Cyclin D1	
High	71 (75)
Low	23 (25)
Not evaluable	17
CD44	
High	22 (24)
Low	70 (76)
Not evaluable	19
Mismatch repair protein	
MSI	7 (8)
MSS	76 (92)
Not evaluable	28

DLL, Delta-like ligand; Jag1, Jagged-1; MSI, Microsatellite instability; MSS, microsatellite stable; NICD, Notch intracellular domain.

### Tissue microarray construction

**Figure 1:**
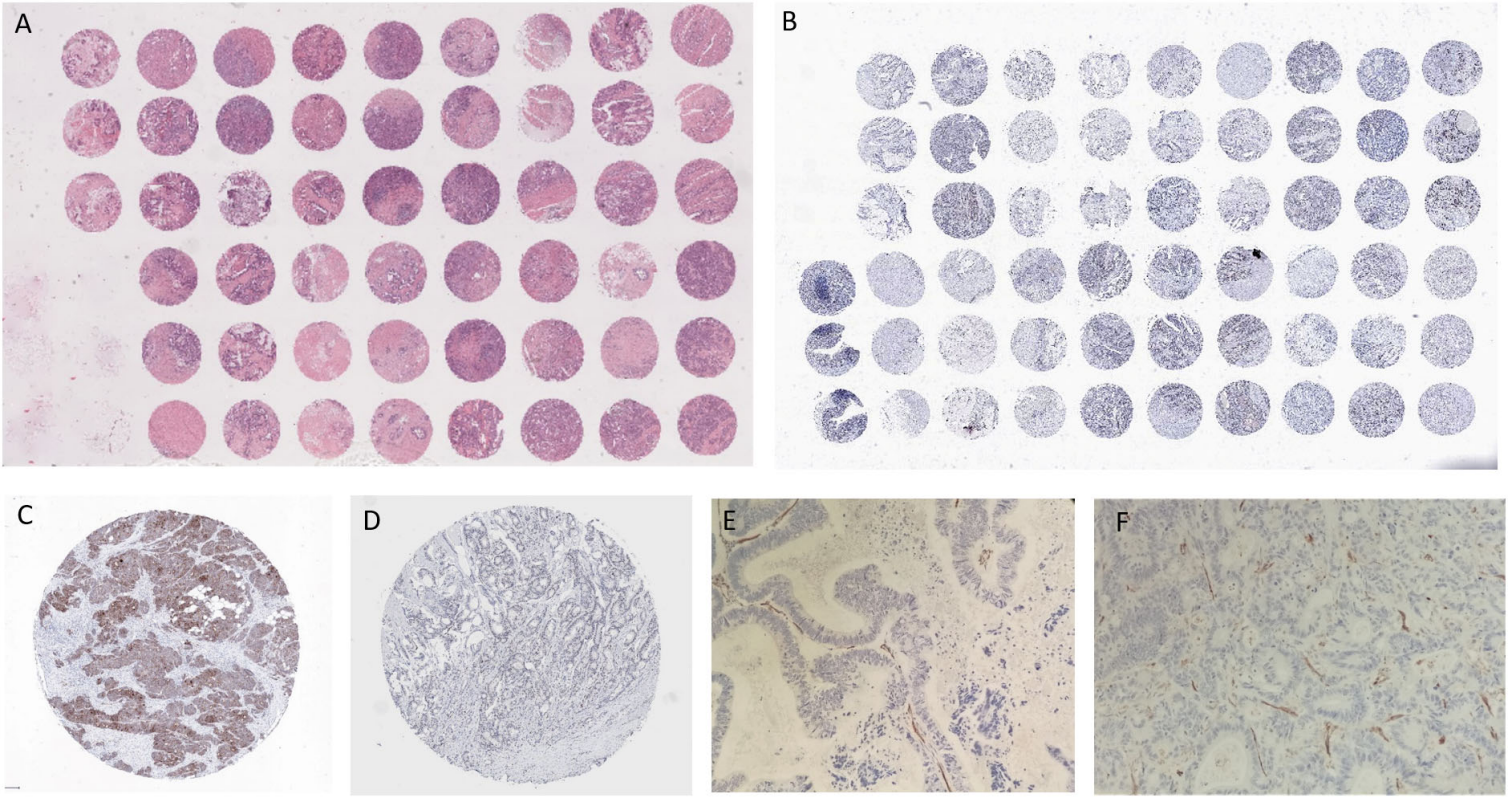
**(A)** TMA, Hematoxylin and Eosin; **(B)** TMA, NICD; **(C)** NICD; **(D)** Jag1; **(E)** CD31 non responder patient; **(F)** CD31 responder patient with NICD and Jag1+.

The following method was used to construct TMAs. Hematoxylin and eosin slides were reviewed to select tumor foci for each patient. A TMA instrument (3DHISTECH) was used to obtain cylindrical tissue cores from the selected areas of each donor block. Cores were assembled and embedded in the recipient block. Each core was 0.6 mm in diameter and its surface measured 0.282 mm^2^ (2 or 3 high-power fields). The distance from one core to the other was 0.7 or 0.8 mm. 5 μm thick sections were cut from the recipient block to perform immunohistochemistry (**Figure 1**).

### Immunohistochemistry

Firstly, the expression of Notch Intracellular Domain (NICD VAL 1744 clone D3B8, dilution 1:100, Cell Signaling Technology), Jag1 (JAG1 clone D4Y1R, dilution 1:100 Cell Signaling Technology) and E-cadherin (clone 36, Ventana Roche, ready-to-use) was studied and only certain staining patterns were considered positive. NICD, Jag1 and E-cadherin staining were considered positive when they showed cytoplasmic and/or nuclear reactivity, cytoplasmic and/or membrane reactivity and membrane reactivity, respectively. Both the intensity and the percentage of stained cells were evaluated. The intensity was assessed as 0 = negative, 1 = weak, 2 = moderate and 3 = strong.

Secondly, the absolute number of CD4+ (clone SP35) and CD8+ (clone SP57) lymphocytes was counted in three different high-power fields and the mean values obtained were used to determine the CD4/CD8 ratio. A CD4/CD8 ratio of 2.0 was considered normal. The assessment of CD3+ (clone 2GLV6), CD44+ (clone SP37) and CyclinD1+ (clone SP4-R) was given as a percentage of positive cells (Ventana Roche, ready-to-use).

Thirdly, the expression of DLL3 (clone SP347, Ventana Roche, ready-to-use) and DLL4 (clone 4A11F8 dilution 1:100 Biorbyt) was studied. Positivity was defined as ≥25% tumor cells, high expression of DLL3/DLL4 was defined as ≥75% tumor cells. The intensity was assessed as 0 = negative, 1 = weak, 2 = moderate and 3 = strong.

Lastly, mismatch repair proteins (MLH1 - clone M1, PMS2 - clone A16-4, MSH2 - clone G219-1129, MSH6 - clone SP93; Ventana Roche, ready-to-use) were studied. Negative expression of one of them was considered proof of microsatellite instability. In fifteen cases we assessed angiogenesis by counting the microvessels at 20x magnification (Nikon, Eclipse E400).

The immunostained sections for CD31 (Ventana, ready-to-use solution) were examined at low power to select the three areas with the highest vascularity (hotspots).

Two pathologists separately assessed each case without any clinical information.

### Radiomic data

Patients that underwent abdominal Computed Tomography (CT) at the University Hospital of Parma for CRC staging were included in the study. CT scans were performed with different CT scanners and imaging protocols; images were retrieved from Picture Archive and Communication System (PACS) and were subsequently imported into a dedicated software (3D Slicer) for tumor segmentation.

One radiologist (ML) evaluated all CT scans visually and identified the target lesion on portal venous phase. The reader was instructed to draw manually multiple regions of interest (ROI) at different levels by tracing the boundaries of the lesions: subsequently, a dedicated tool (SlicerRadiomics) software interpolated the ROIs to obtain the volume of interest (VOI) which allowed the extraction of 852 radiomic features (RF). The VOI was manually modified by the reader in case of inaccurate segmentation. Image preprocessing based on wavelet decomposition was performed by SlicerRadiomic before feature calculation to generate independent radiomic predictors.

The radiomic dataset included shape, first-order, Gray-Level- Co-occurrence-Matrix (GLCM), Gray-Level-Run-Length-Matrix (GLRLM), Gray-Level-Size-Zone-Matrix (GLSZM), Neighboring-Gray-Tone-Difference-Matrix (NGTDM), Gray-Level-Dependence-Matrix (GLDM).

## Statistical analysis and classification model

### Classical statistics

The chi-square test and Fisher’s exact test were used to perform univariate comparisons between categorical variables.

The Kaplan-Meier method was used to estimate the mean and median time for progression free survival (PFS) followed by a Cox regression analysis to evaluate the relationship between survival and covariates in a multivariable framework. The model was evaluated by making use of model diagnostics. This included checking for the overall goodness of fit, model adherence to key assumptions, influential observations and nonlinearity. The variables considered in the Cox regression were KRAS, type of chemotherapy protocol, site of primary tumor, NICD, CD44, Jag1, CD3, DLL4 expression; only NICD expression resulted statistically significant and was maintained in the final model. The regression coefficients were reported as hazard ratios (HRs). The 95% confidence intervals (CIs) were also estimated from the analysis.

The commercial package IBM-SPSS v.28 and the open-source statistical system Jamovi version 2.3.0, which is based on the widely used open-source system R, were used to perform survival analysis. A p-value less than 0.05 was considered statistically significant (p < 0.05).

### Multiomic models

Classification models were developed to predict time to disease progression. With this aim in mind, PFS at 9 months was used to stratify patients in two groups, namely short and long-term survivals. We considered PFS at 9 months as a target variable because a comprehensive meta-analysis has recently showed that PFS ranges between 7 and 10.8 months for CRC patients treated with bevacizumab (18). Therefore, we acquired the central value of that interval from the meta-analysis to further stratify the prognosis of our patients according to the integrated profile. Three models were developed: radiomic (R), clinical/Notch signaling (C/N) and the comprehensive integrated model (I). In the R model, we removed redundant highly correlated features by calculating their Spearman Rho correlation coefficient: RFs with a coefficient greater than 0.99 were excluded from the successive analyses. Subsequently, feature standardization by z-score was applied. In the C/N model, the same variables considered in the Cox regression were added as predictors (**Table 1**). In both R and C/N models, a L2 penalized logistic regression algorithm was implemented for features selection and validation. Most predictive features were selected by means of a wrapper approach, i.e. the sequential forward feature selection algorithm with 20 Monte-Carlo cross-validation (MCCV) splits. We chose the area under the receiver operating characteristic (ROC) curve (ROC-AUC) as performance metric. We iterated the selection process 50 times, using 50 different random states and, subsequently, we selected the features that had higher frequency of occurrence for both R and C/N models. Through 5000 MCCV splits (train:0.7, test:0.3), different numbers of clinical/genomic and radiomic features were selected and used respectively for R and C/N model training and validation. Likelihood ratio test was applied to verify if the addition of another feature significantly improved the model performance. The selected C/N and R features were used together to build the I model.

For all models, the ROC-AUC and accuracy scores for each MCCV were calculated and averaged over these iterations. Mean ROC curve and mean learning curve were also plotted. Recall and precision metrics were also calculated

Survival analysis was performed in radiomic dataset. Kaplan–Meier survival curves PFS for two risk groups were calculated and then compared using log-rank test. The risk groups were assessed by using continuous RFs, previously selected by the machine-learning model. Risk groups based on RFs were developed using ROC analysis to determine the cutoff value of each RF for optimal stratification into two classes: Youden index was chosen as optimal threshold. Subsequently, we combined the selected features in a single variable and we performed Kaplan–Meier analysis again. Finally, we calculated the probability to predict longer-term class in each risk group of combined features. Probabilities derived from the R model were averaged over MCCV splits.

Machine-learning model, analysis and plots were performed by means of Python v. 3.8.5; scikit-learn and MLextend machine learning libraries were used for features selection and model development.

## Results

### Classical statistics

A total of 111 patients have been included in the analysis. The cohort is shown in **Table 1**. A positive association was found in univariate analysis between NICD and Jag1 expression (p < 0.001; **Table 2**). No significant association was found for the other analyzed markers and KRAS mutation (data not shown). All main clinical characteristics were comparable among the subgroups of patients (data not shown). Specifically, no significant associations of NICD and Jag1 immunostaining scores with age, baseline CEA levels, number of metastatic sites and subsequent chemotherapy were observed.

**Table 2:** Association between NICD and Jag1 expression in CRC.

	Low Jag1	High Jag1	
**Low NICD**	38 (56%)	30 (44%)	
**High NICD**	4 (10%)	38 (90%)	
			χ² continuity correction p < 0.001
			Fisher’s exact test p < 0.001

Jag1, Jagged-1; NICD, Notch intracellular domain.

**Figure 2:**
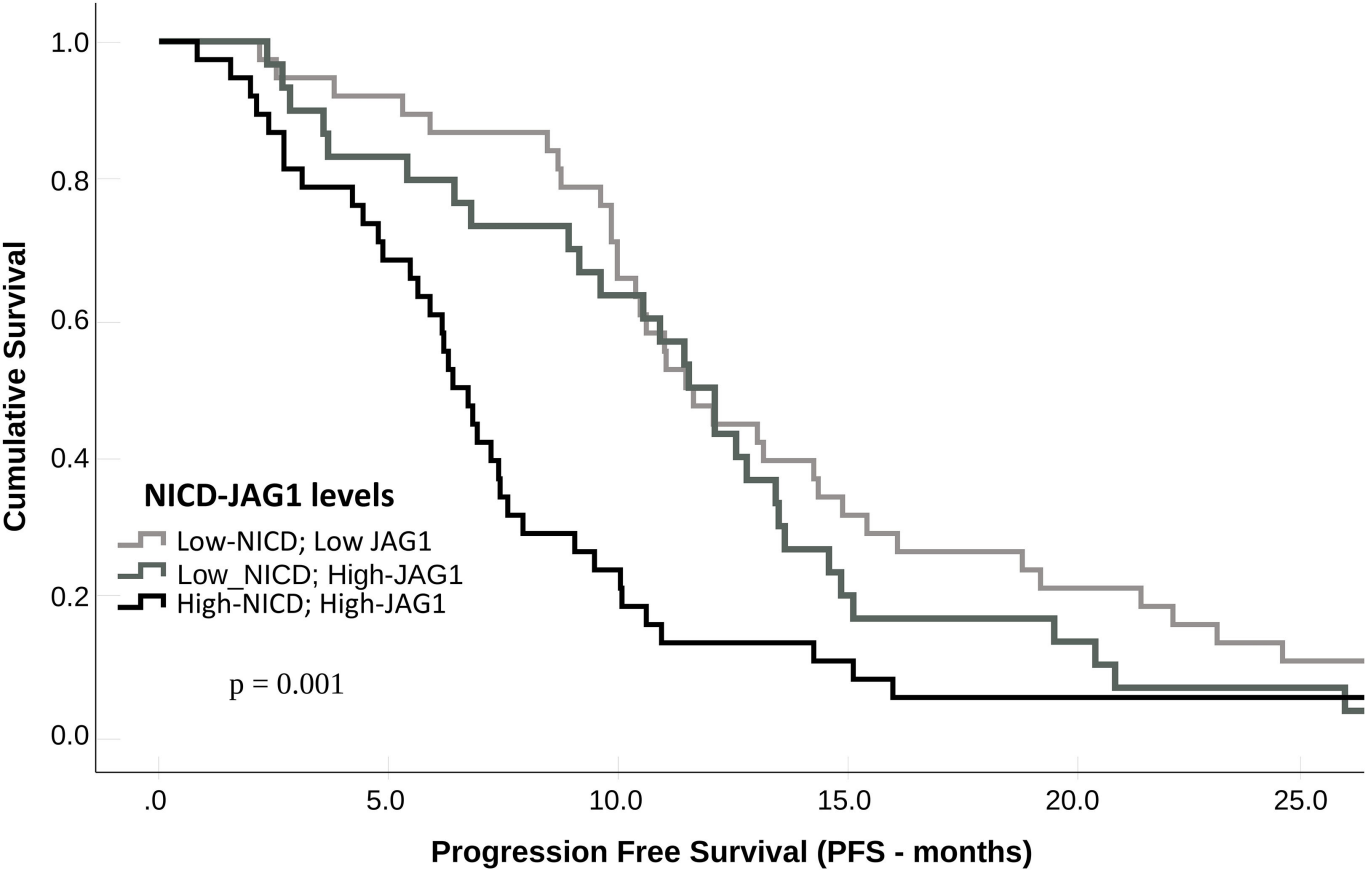
Progression-free survival (PFS) according to NICD and Jag1 expression levels in metastatic CRC patients treated with bevacizumab.

Compared with patients who had NICD and/or Jag1 low tumors, patients whose pre-treatment tumors expressed high NICD and Jag1 levels showed poor RECIST 1.1 categories with higher rates of stable disease (SD) or progressive disease (PD) as best response, and lower frequencies of complete response (CR) or partial response (PR); p = 0.002 (**Table 3**). Associations between NICD and Jag1 and therapy response were further evaluated using PFS and Kaplan-Meier and Cox proportional hazard modeling. Median PFS was significantly shorter in patients whose tumors expressed high NICD and Jag1 (6.43 months vs 11.53 months for negative cases; p = 0.001, **Figure 2**). Cox regression following univariate analysis confirmed NICD as the only independent predictor for PFS (HR = 1.820 [1.165 – 2.844]; p = 0.009).

**Table 3:** Response according to NICD and Jag1 protein expression.

	Response rate		Total
NICD-Jag1	SD + PD	CR + PR	
Low NICD_Low Jag1	13 (34%)	25 (66%)	38
Low NICD_High Jag1	21 (70%)	9 (30%)	30
High NICD_Low Jag1	3 (75%)	1 (25%)	4
High NICD_High Jag1	28 (74%)	10 (26%)	30
			χ² continuity correction p = 0.002
			Fisher’s exact test p < 0.001

CR, complete response; Jag1, Jagged-1; NICD, Notch intracellular domain; PD, progressive disease; PR, partial response; SD, stable disease.

Quite surprisingly, 5 patients with high NICD tumors showed long PFS. Each case was evaluated for the following features: inflammation, staging, grading and microvascular density. The last one was the only noteworthy characteristic. For this reason, we assessed the microvascular density according to Chalkey’s methods: microvessels were counted manually for each hotspot at 20x magnification (high power field) and expressed as MVD score. This assessment was carried out for 15 patients based on response to therapy: 5 were non responder (NICD/Jag1 positive), 10 were responder (5 NICD/Jag1 positive and 5 NICD/Jag1 negative). Those with an MVD score ≥5 (CD31-high, NICD/Jag1 positive) were associated with significantly poorer survival. Low CD31 was seen in all 10 responder patients (both 5 NICD/Jag1 positive and NICD/Jag1 negative) and associated with a better prognosis.

### Multiomics

The retrospectively collected 111 CRC cases were decreased due to inclusion criteria that comprised the availability of (i) CT data and (ii) PFS information. Thus, the ensuing results based on the multiomic approach refer to a restricted population of 76 subjects. Regarding feature preprocessing, the Spearman correlation matrix for RFs is reported in **Figure S1**. Redundant features were removed, thereby reducing the number of RFs by about 33.6%.

In the R model, the best performance in differentiating short and long survival was obtained by selecting two RFs: Strength (NGTDM) and Skewness (first order) with a ROC-AUC of 0.709 and an accuracy of 0.671. Likelihood ratio showed that the performance of the model would not significantly improve by adding further RFs (**Table 4**).

**Table 4:** Performances of the R, C/N and I models.

Model name	ROC AUC	ROC AUC 95%CI	Accuracy	Accuracy 95%CI
**R**	0.709	0.706-0.711	0.671	0.668-0.673
**C/N**	0.743	0.741-0.745	0.649	0.647-0.651
**I**	0.823	0.824-0.828	0.751	0.749-0.753

In the C/N Model, the most predictive features were Jag1 and NICD, whereas the addition of a third feature was not significantly relevant: the ROC-AUC was higher as compared to the R model alone, with a ROC-AUC of 0.743, while accuracy was slightly lower (0.649) (**Table 4**).

**Figure 3:**
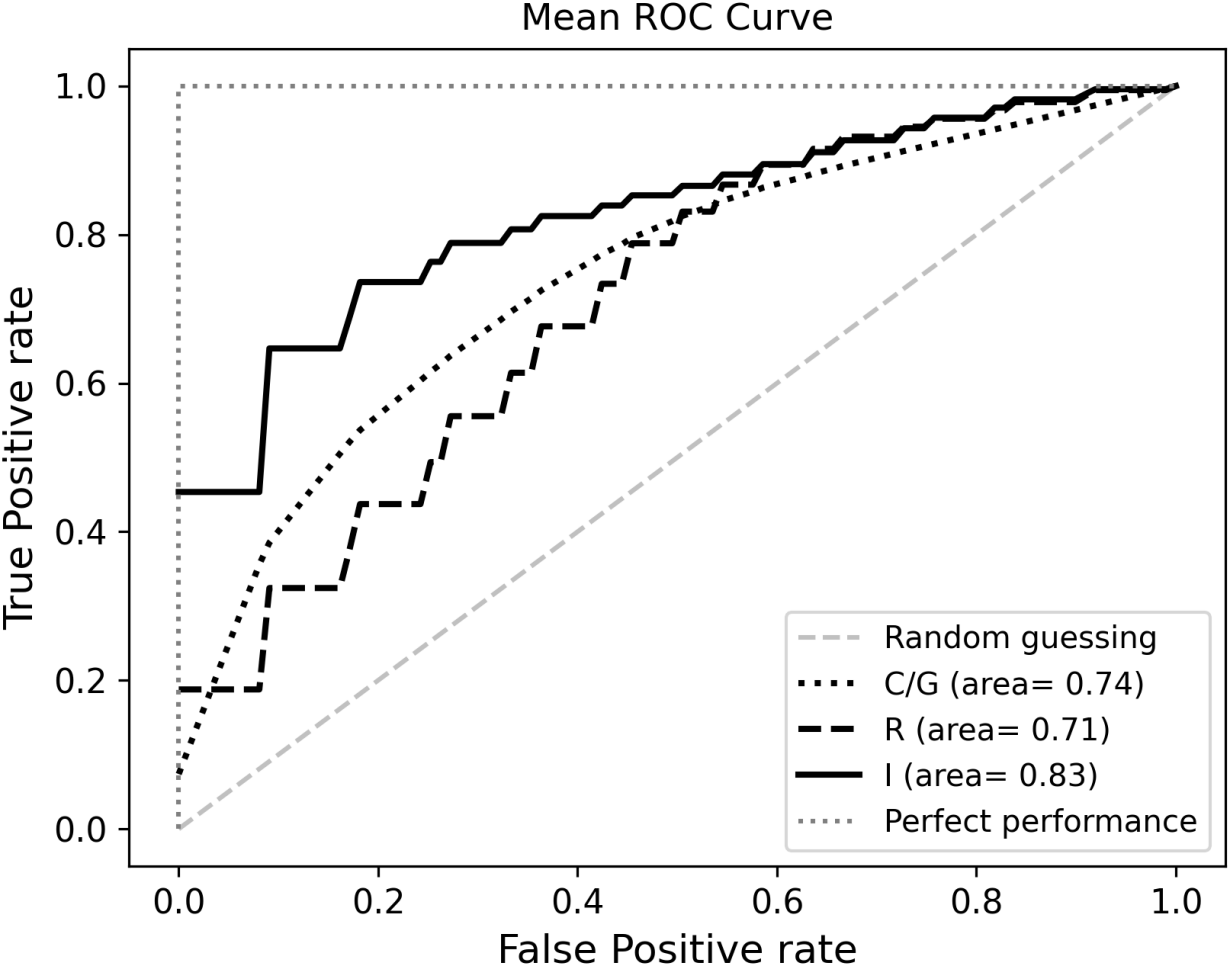
Mean receiver operating characteristic (ROC) curves.

The I Model included the previously selected R and C/N features, that had a negative effect on the likelihood of predicting long survivors, as shown by odds ratios (**Table S1**). The I model yielded the highest ROC-AUC (0.823) and accuracy (0.751) values. The mean ROC curves are displayed in **Figure 3**.

Learning curves show the ROC-AUC score (**Figure S2A**) and accuracy (**Figure S2B**) as a function of the number of training samples. We plotted performance scores obtained by predictions for both training (blue line) and validation (green line) datasets and averaged over all iterations of the MCCV. For each model, we also calculated and averaged over 5000 MCCV splits the recall metric representing the true predictions of longer survival class: 0.794 [95% CI : 0.790, 0.797] for R model, 0.660 [95% CI: 0.654, 0.666] for C model and 0.767 [95% CI : 0.763, 0.770] for I model. In addition, we calculated precision metrics (i.e. positive predictive value), representing the fraction of true positive cases among the total positive predicted instances: 0.642 [95% CI : 0.641, 0.645] for R model, 0.673 [95% CI: 0.669, 0.676] for C model and 0.751 [95% CI : 0.749, 0.754] for I model.

**Figure 4:**
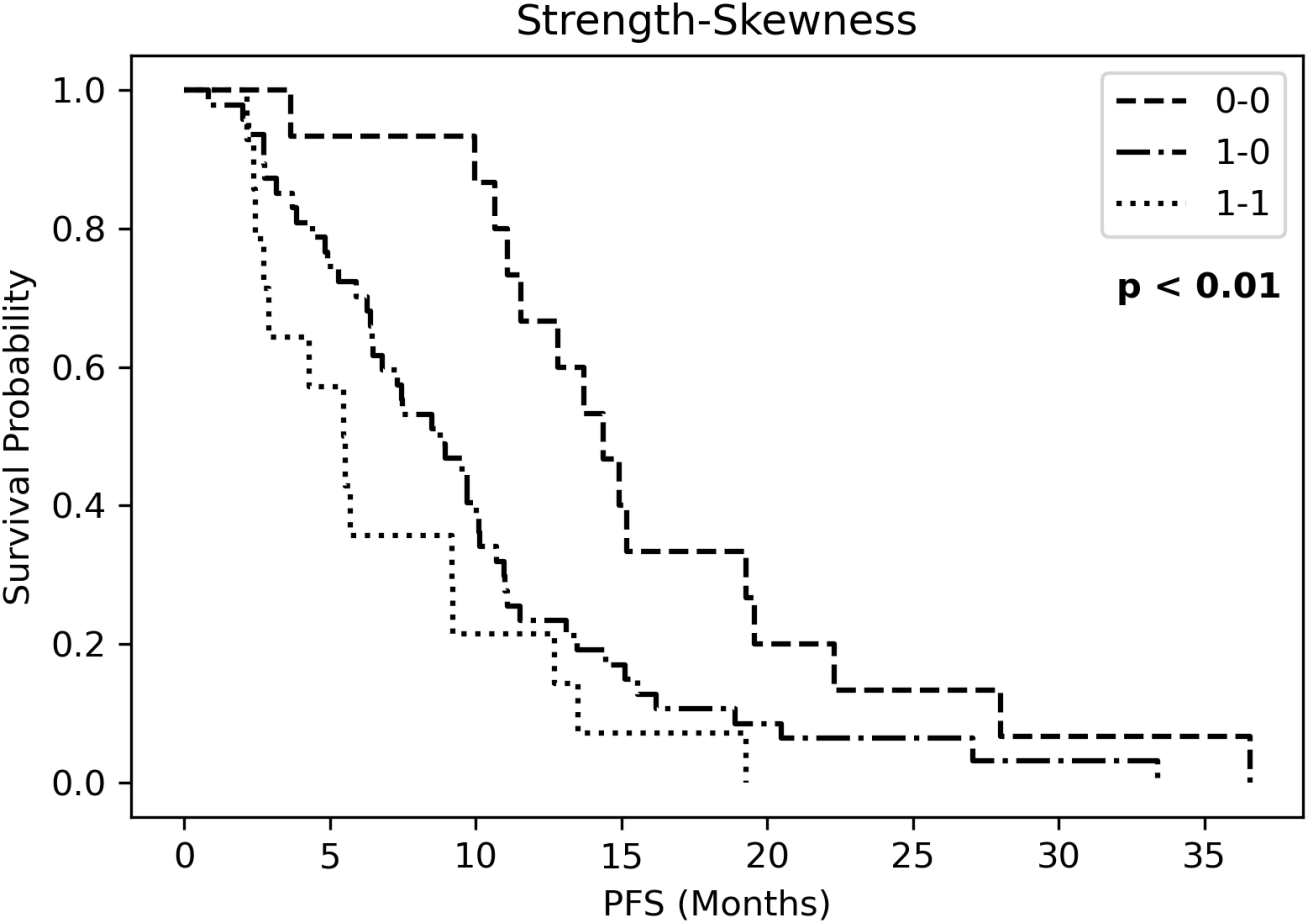
Kaplan Meier curves of progression-free survival (PFS) for three risk groups identified by Strength-Skewness (ST, Strength; SK, Skewness).

Kaplan Meier curves of PFS (**Figure S3**) showed significantly different risk strata for Strength, whereas none for skewness. The combined RF (strength-skewness) created 3 risk groups which significantly stratified in PFS curve (**Figure 4**). Probabilities of longer-term class prediction are listed in **Table 5**.

**Table 5:** Probabilities of longer-term class prediction.

R MODEL			
Strength	Skewness	Probability	Standard deviation
0	0	0.692	0.024
1	1	0.300	0.029
0	1	0.498	0.028

## Discussion

The diagnosis of CRC is based on the integration of multiple features (histopathology, immunohistochemistry and molecular findings) and its management is of the utmost importance. Although immunohistochemistry has been widely used to detect microsatellite instability in CRC screening for defective DNA mismatch repair, unexpectedly negative results have been reported probably due to somatic mutations. This implies that the analysis should be completed with microsatellite instability-polymerase chain reaction test to have reliable results (19).

In this study, we investigated NICD expression and a series of other correlated markers that have been previously associated with angiogenesis to predict tumor progression-free in advanced stage CRC treated with bevacizumab and first-line chemotherapy. Our results show that high NICD and Jag1 expression are associated with PD and early disease progression to anti VEGF-based therapy.

Notch signaling may regulate both the initiation and the cessation of angiogenesis through different mechanisms (20). The potentiality of Notch signaling to rule angiogenic processes becomes crucial in the context of aberrant angiogenesis. Furthermore, neoangiogenesis in CRC may differ in distinct tumor subtypes (21).

Angiogenesis is the expansion of emergent vascular sprouts from preexisting blood vessels. Luminal endothelial cells switch into tip cells that lead to the outgrowth of a multicellular stalk. Notch signaling involves cell fate determination as a mechanism to determine tip and stalk cells (21). The distribution of vascular sprouts depends on Notch triggering; moreover, the formation of a new sprout or the alteration of the original vessel relies upon Notch-DLL4 expression in endothelial tip cells (20). VEGF signaling can be downregulated in cells with activated Notch signaling by decreasing VEGF receptor transcription levels (22–24). In these cases, the uncontrolled dysfunctional tumor vessels proliferation under Notch signaling is not inhibited by VEGFR. The uncontrolled angiogenesis increases tumor hypoxia which is detrimental to chemotherapy as well. VEGF regulates blood vessel function by inducing tumor cell growth and suppressing immune activation (25).

Unlike DLL4, Jag1 is overexpressed in tumor cells. It is supposed to work as a communication element between tumor cells and tumor-associated endothelial cells to trigger Notch signaling, enhance cell proliferation and stabilize vessels (26). Jag1 is a critical regulator of tip cell formation and sprouting because of its ability to modulate DLL4-Notch signaling in the angiogenic endothelium (20). Notch and VEGF induce the expression of DLL4 (27, 28); on the contrary, Jag1 is not upregulated by Notch and is induced by inflammatory cytokines, such as TNF-α, which reduces DLL4 transcription. These signals might modulate angiogenesis by changing the ratio of DLL4 and Jag1 expression, allowing the integration of different pro or antiangiogenic signals. The intricate interaction of the ligands DLL4 and Jag1 traces the pathway of tip cell selection (20).

Although the detailed mechanisms behind Notch activation have not been fully discovered, it is known that the related soluble ligands influence several contexts. They regulate the proliferation of regulatory T cells (7, 9), influence tumor microenvironment, promote adipocyte differentiation (29), mediate hematopoietic cell differentiation (30) and neurogenesis (31). Moreover, Jag1 overexpression in cancer cells can activate Notch signaling in adjacent endothelial cells (32). Our study focused on NICD expression, however did not underestimate the role of tumor microenvironment. In fact, the assessment of CD3 and CD4/CD8 ratio did not show a correlation with tumor aggressiveness or survival. Although we have restricted our analysis to lymphocytes, we know that Notch signaling can also affect other factors such as tumor associated fibroblasts, endothelial cells and the expression of CTLA4 in tumor infiltrating lymphocytes. Further studies are necessary to assess the interaction of Notch with other stromal cells (33, 34).

This study did not prove that DLL4 was relevant to define the biological behavior of tumors. Patients with a highly vascular tumor microenvironment went worse in comparison to those with a poor tumor vascularization. However, the expression of Notch and Jag1 was associated with a better outcome only in those patients with a poor tumor vascularization.

We developed an integrated model which included clinical, genomic and radiomic variables to explore its potential role in the prediction of survival. The model was designed to predict 9-months PFS in CRC patients and included a first-order (skewness) and a second-order (NGTDM strength), along with NICD and Jag1 expression levels. Results showed that each additional increase of one point of NGTDM strength - which accounts for tumor heterogeneity - was associated with approximately 50% decrease in the odds of survival.

Regarding prognostic performance, our radiomic model agreed with other CT-based radiomic models that have been proposed, which yielded ROC-AUCs between 0.66 and 0.74 (18, 35). The integration of radiomics with clinical (36) and genomic predictors have led to increased model performances (17, 37, 38); this study has confirmed this finding. Cao et al. tested a radiomic signature in 381 patients with CRC and showed that a radiomic-derived score was able to stratify their outcome and enrich the TNM staging (39). In our study, we differentiated three groups of patients based on binarization of the values of RFs: those individuals with lower RF “skewness” were those with longer survival; similarly, when patients displayed lower RF “strength” values their outcome was better. Lower “skewness” and “strength” values might potentially be related to more homogeneous lesions, which could be related to a more favorable outcome. Radiomics might represent a step forward into personalized and tailored medicine, helping to identify patients that might benefit most from therapy.

Our research has some limitations. Firstly, we enrolled a single center retrospective cohort and no external validation was considered: performances of our multiomic models derived from cross-validation analyses. Therefore, further studies based on datasets from other centers are needed to evaluate model generalizability. Secondly, the repeatability and robustness of radiomic features with respect to CT acquisition parameters and to manual segmentation were not addressed. We recognize the reproducibility of manual segmentations of CRC to be a potential source of variability potentially affecting the results, given the potential challenges in the identification of the boundaries of such lesions with an impact on the generalization of our model. Therefore, we look forward to future studies on larger populations with multiple readers to be involved in the segmentation process. However, the purpose of the radiomic analysis of this study was to produce preliminary results to be compared with the histopathological data.

In conclusion, this study provides the first evidence that high NICD and Jag1 expression predict early disease progression in CRC patients treated with anti-VEGF-based therapy.

Although the data must be confirmed in a larger series, the increase in intratumoral microvascular density could predict a lower response to treatment.

Further studies will be necessary to demonstrate our hypothesis that newly formed vessels in tumors expressing elevated NICD do not benefit from bevacizumab and expand our preliminary results on the potential role of radiomics to improve the prediction of outcome of CRC patients.

## Data availability statement

The data that support the findings of this study are available on request from the corresponding author, [FN].

## Ethics statement

The studies involving human participants were reviewed and approved by AVEN: Comitato Etico dell’Area Vasta Emilia Nord. The patients/participants provided their written informed consent to participate in this study.

## Author contributions

FN, LG, RS and LB conceived the study. LG, NC, ES, GM, LL, MM, CB, AZ and GP performed the analyses. FN, LG, LB, GP, LL, GM, MM, RS, ES, CA, GLdA interpreted the data, and wrote the manuscript. GDR and FG helped to review the manuscript. All authors contributed to the article and approved the submitted version.
