# Supplementary material for: Notch-Jagged1 signaling and response to bevacizumab therapy in advanced colorectal cancer: A glance to radiomics or back to physiopathology?
Source: Front Oncol. 2023 Feb 28;13:1132564. doi: 10.3389/fonc.2023.1132564 (PMC10011088; doi:10.3389/fonc.2023.1132564)

Supplementary material

Table S1. Odds ratio

| **Feature name** | **Odds ratio** | **95% CI** |
| --- | --- | --- |
| **NGTDM-Strength** | **0.47** | **0.35-0.61** |
| **Firstorder-Skewness** | **0.54** | **0.36-0.73** |
| **NICD** | **0.43** | **0.30-0.61** |
| **Jag1** | **0.43** | **0.31-0.59** |

Figure S1. Spearman correlation matrix.


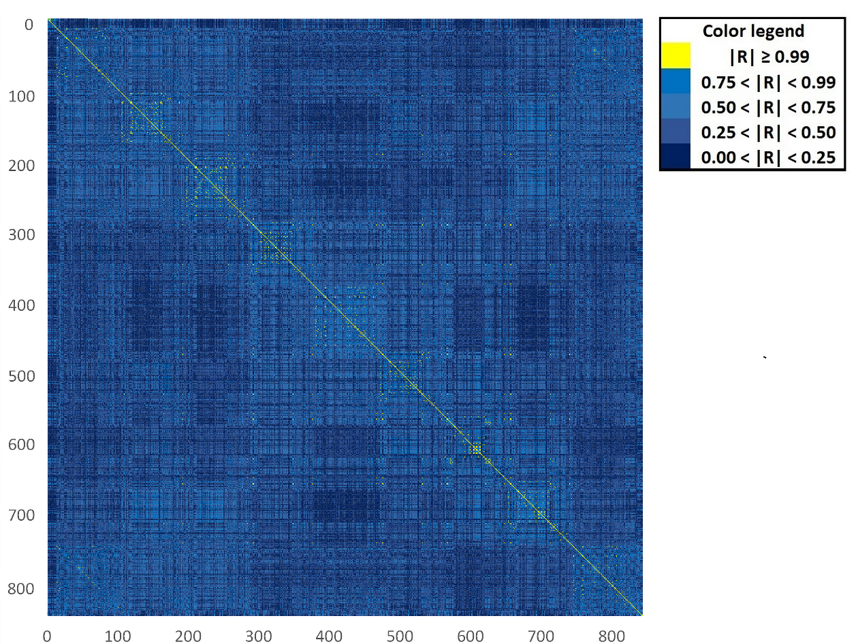


Figure S2. Learning curves of ROC AUC (A) and Accuracy (B).


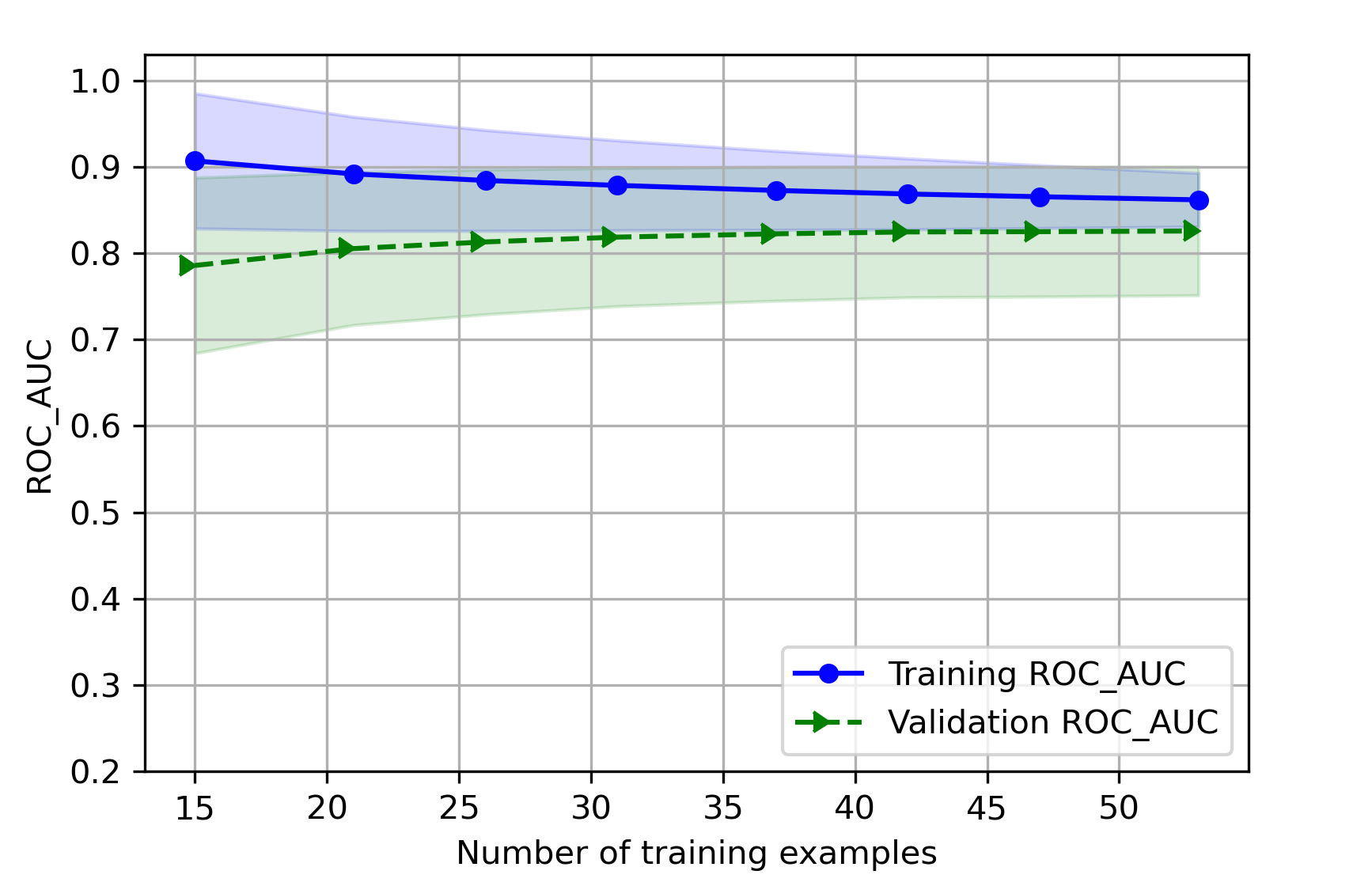


A


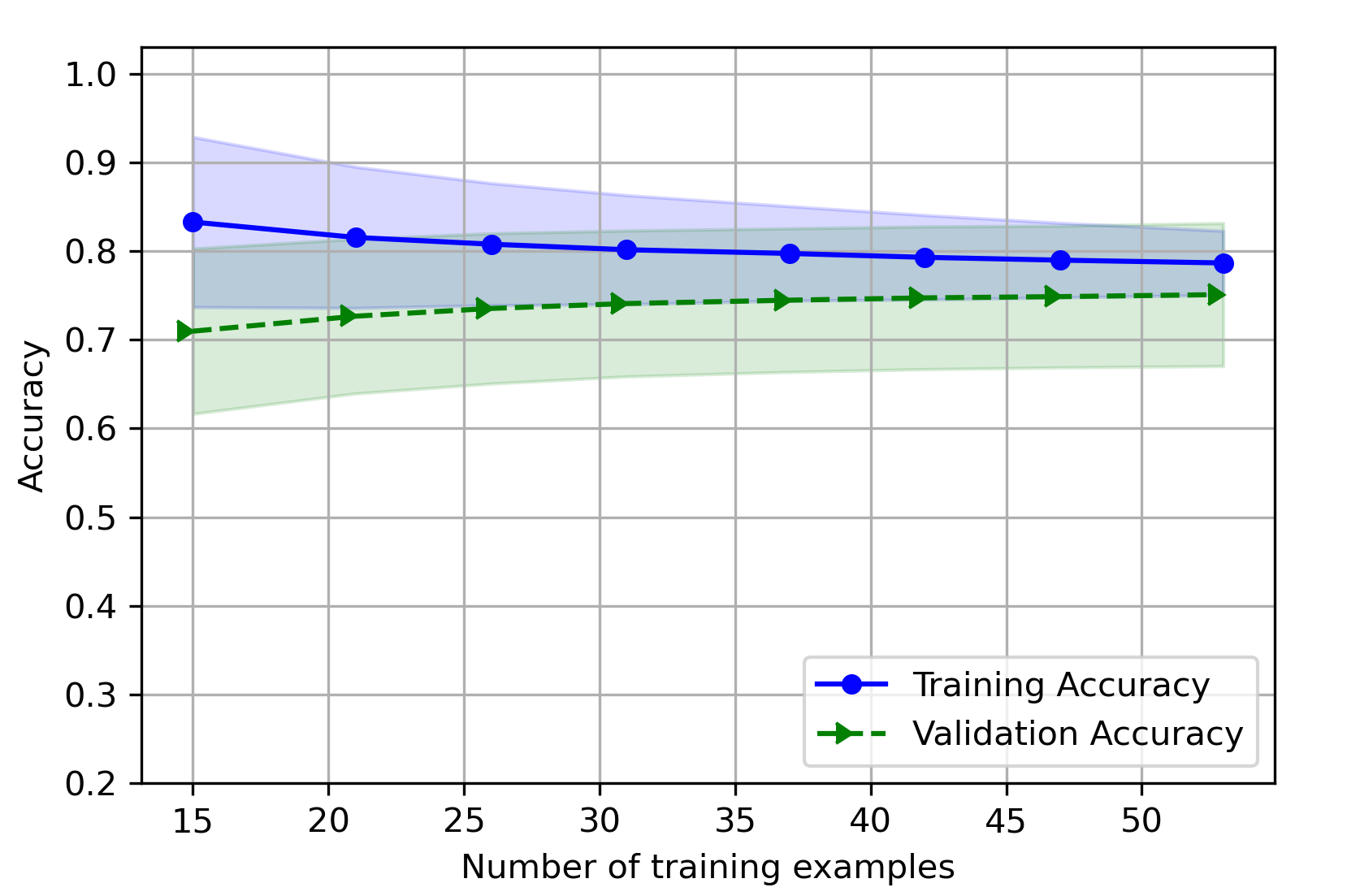


B

Figure S3. Kaplan Meier curves of PFS for two risk groups identified by continuous RF categorized by Youden index: Strength (A) and Skewness (B). These plots showed significantly different risk strata for Strength, whereas none for skewness.

A


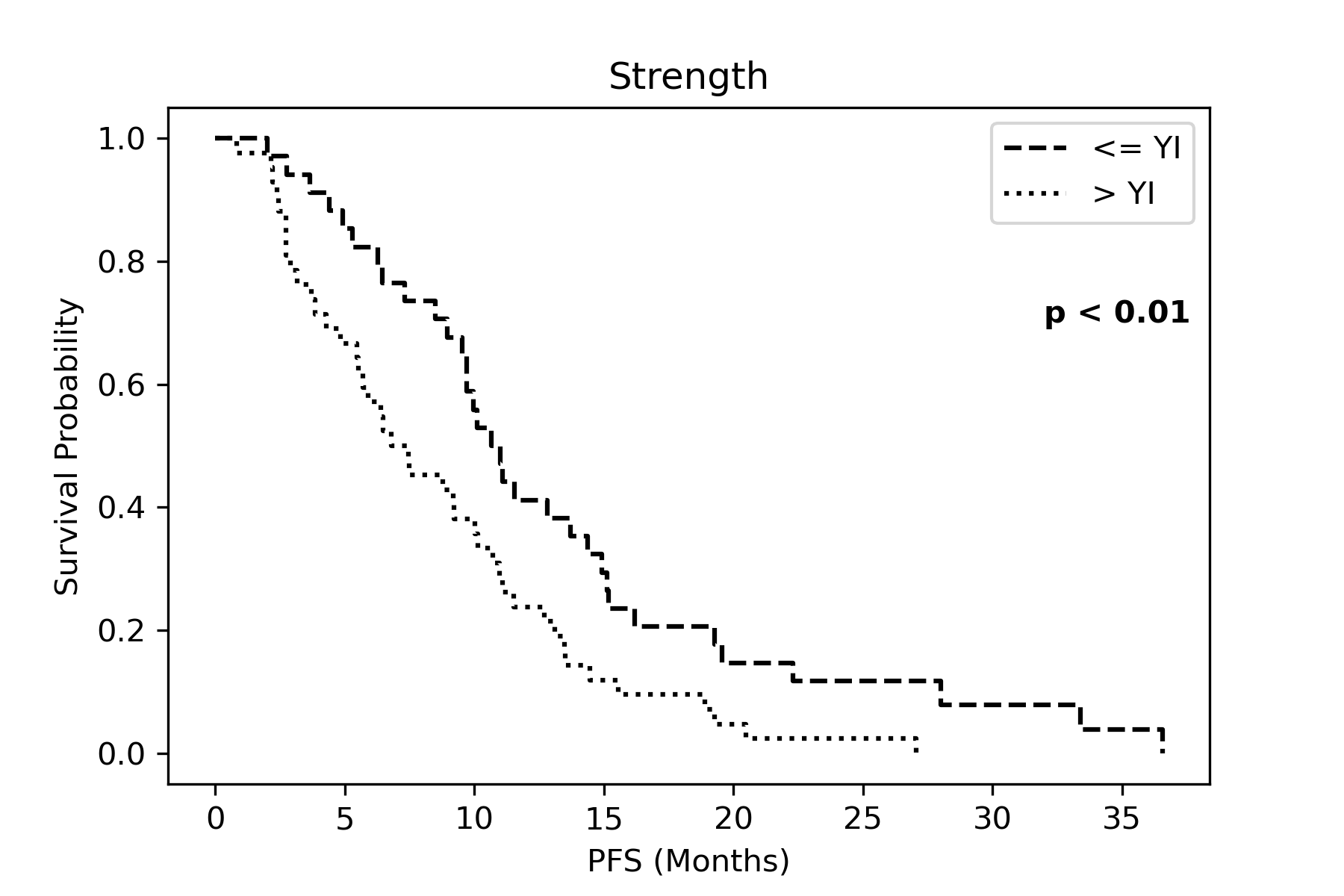


B


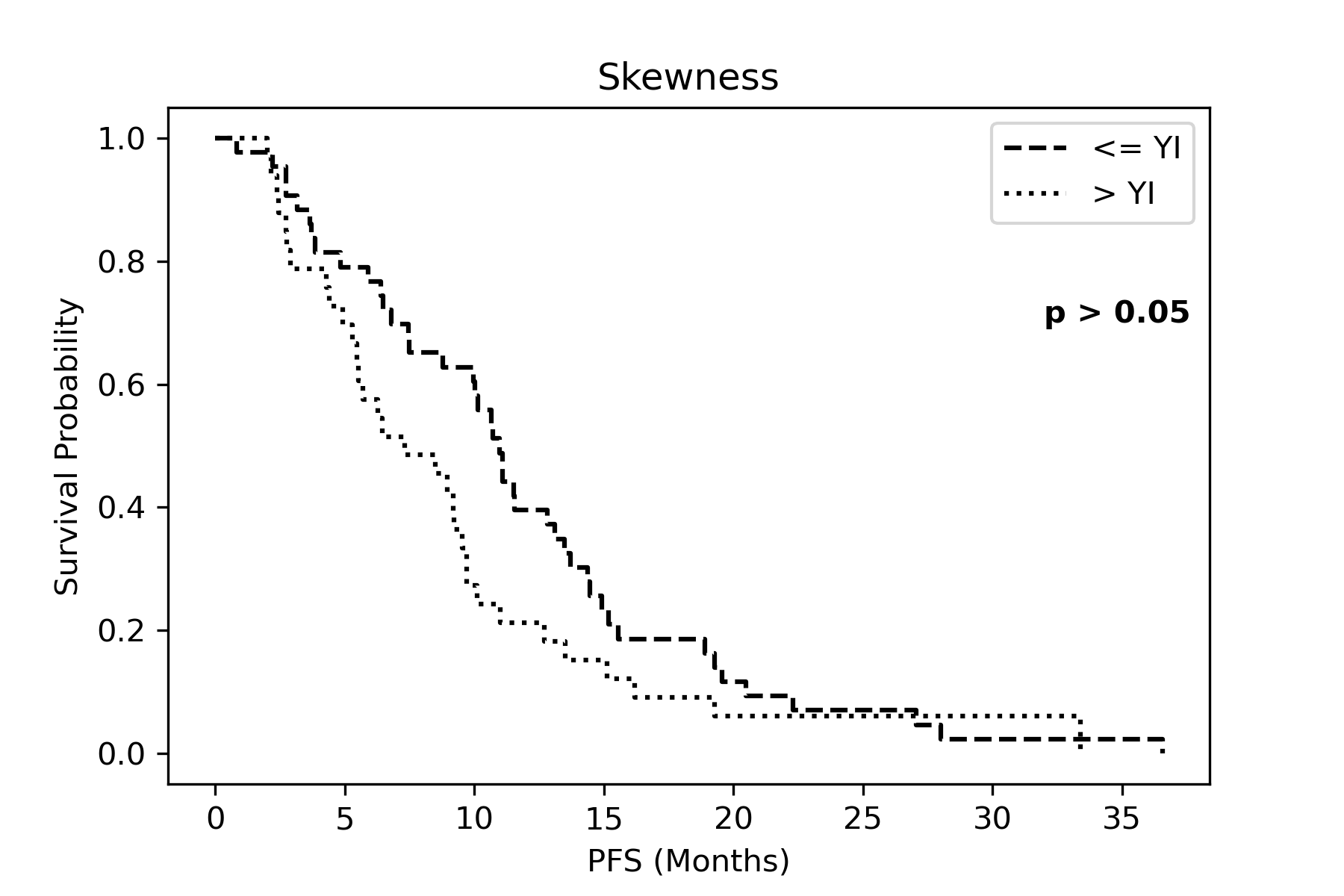

Supplement: Supplementary file 1 [file DataSheet_1.docx]
